# Progesterone Receptor B signaling Reduces Breast Cancer Cell Aggressiveness: Role of Cyclin-D1/Cdk4 Mediating Paxillin Phosphorylation

**DOI:** 10.3390/cancers11081201

**Published:** 2019-08-17

**Authors:** Francesca Ida Montalto, Francesca Giordano, Chiara Chiodo, Stefania Marsico, Loredana Mauro, Diego Sisci, Saveria Aquila, Marilena Lanzino, Maria Luisa Panno, Sebastiano Andò, Francesca De Amicis

**Affiliations:** 1 Centro Sanitario, University of Calabria, Via P. Bucci, 87036 Rende, Italy; 2 Department of Pharmacy, Health and Nutritional Sciences, University of Calabria, Via P. Bucci, 87036 Rende, Italy

**Keywords:** invasion, EMT, luminal A, paxillin, Rho GTPase

## Abstract

Progesterone-Receptor (PR) positivity is related with an enhanced response to breast cancer therapy, conversely cyclin D1 (CD1) is a retained marker of poor outcome. Herein, we demonstrate that hydroxyprogesterone (OHPg) through progesterone receptor B (PR-B) reduces breast cancer cell aggressiveness, by targeting the cytoplasmic CD1. Specifically, OHPg diminishes CD1 expression by a transcriptional regulation due to the recruitment of PR-B at a canonical half-PRE site of the CD1 promoter, together with HDAC1, determining a chromatin conformation less prone for gene transcription. CD1, together with its kinase partner Cdk4, regulates cell migration and metastasis, through the association with key components of focal adhesion, such as Paxillin (Pxn). Kaplan-Meier analysis shows that low Pxn expression was associated with increased distant metastasis-free survival in luminal A PR+ breast carcinomas. Interestingly, OHPg treatment reduced Pxn content in T47-D and MCF-7 cells; besides, the interaction between endogenous cytoplasmic CD1/Cdk4 with Pxn was reduced. This was consistent with the reduction of *p*-Ser83Pxn levels, crucially causing the delay in cell migration and a concomitant inhibition of Rac1 activity and *p*-PAK. Collectively, these findings support the role of PR-B in breast epithelial cell integrity and reinforce the importance in targeting PR-B as a potential strategy to restrict breast tumor cell invasion and metastasis.

## 1. Introduction

Breast cancer is the most frequent kind of cancer, and the second cause of cancer mortality among women in many developed countries [1]. A large amount of breast cancers are sporadic and attributable partially to long-term exposure to estrogens, driving growing genetic and epi-genetic changes, and consequent progressive carcinogenesis of breast cells. The latter arises from an early non-tumorigenic pre-malignancy to the late malignant tumorigenic steps [2].

Breast cancers are typically assessed for Estrogen Receptor α (ERα), but also Progesterone Receptors (PR-B and PR-A) and HER2 expression are used to define histological subtype and guide treatment options. Besides, cyclin D1 (CD1) is retained a marker of poor prognosis. Specifically, CD1 overexpression has been associated with breast cancer metastasis in clinical studies [3], while CD1-deficient cells showed a reduced metastatic potential in vitro [4]. CD1 is a known oncogene modified, both in an inhibitory and stimulatory manner, by the activity of multiple members of the steroid hormone receptor family of nuclear receptors [5].

On the other hand, overexpression of CD1 increased ER α activity, via recruitment of steroid receptor coactivator (SRC1) to estrogen response elements in the absence of ligand [6], while similar experimental conditions failed to alter PRs’ transcriptional activity as measured using reporter gene readouts [7]. However, the precise cellular mechanisms through which aberrant CD1 expression drives breast carcinogenesis and progression are still less well established.

It is widely discussed concerning the role of CD1, together with its binding partner Cdk4, as being an essential regulator of G1 to S-phase transition [8], besides, emerging evidence suggests that CD1 might act through pathways that do not involve its widely accepted function as a cell cycle regulator [9]. Specifically, CD1 has been implicated in various activities, such as chromosomal instability, mitochondrial function, cell adhesion and invasion [9,10]. Thus, the best studied role of CD1 in breast tumorigenesis is the regulation of transcription into the nucleus, although some current studies have also suggested a cytoplasmic function.

Mostly, it is evidenced concerning the physical and functional interaction of CD1 with cytoplasmic and membrane-associated proteins, such as filamin A, RhoA, Ral GTPases and paxillin (Pxn), showing the action of the cyclin in the cytoplasm, influencing adherence and migration [11,12]. Recently, an elegant study demonstrates that the localization of CD1 in the membrane of fibroblasts and tumor cells is decisive for cell migration and invasion [13].

Metastatic invasion is the primary cause of patient mortality related to breast cancer progression. A low invasive potential is related to ER/PR positivity in vitro [14,15]. Accordingly, a recent important study demonstrates functional significance of these steroid receptors crosstalk, dictated by PR, through the regulation of a gene expression program associated with good clinical outcomes [16]. Moreover, clinical studies evidence that high levels of PR correlate with decreased metastatic events in early stage disease [17], while ER-positive/PR-negative breast carcinomas are associated with worse long-term outcomes and metastases after neoadjuvant therapy [18]. Multivariate analyses including patients treated with tamoxifen and aromatase inhibitors demonstrate that PR status is independently associated with being disease-free and overall survival [19]. Moreover progesterone injection preceding to surgery can offer a clinical benefit [16]. Consistently, the results of a multi-institute cohort study indicated that endocrine treatment could not prevent distant metastasis in PR-negative breast cancer patients [16]. Thus, PR is frequently expressed in breast tumors, and may serve as a predictive marker, even if molecular events following PR activation and leading to modulation of cell invasion in breast cancer cells are still debated [20]. Particularly PR action in mediating progesterone effects is highly context dependent [21] and could depend on the Progesterone dose. Wang et al. 2016 [22] demonstrate that high doses of Progesterone activate the cSrc/AKT signaling pathway, preventing RhoA degradation and eventually enhanced migration. Moreover, only a limited number of studies investigate the specific role of the natural ligand Progesterone [23], most concern synthetic Progestins which are known to be endowed with some non-progesterone-like effects, due to nonspecific binding with others steroid receptors [24,25,26]. Taken together, all these published data indicate the need of a deeper investigation of the PR role as a mechanistic player in breast cancer progression.

Herein, we demonstrate that PR-B activation by hydroxyprogesterone (OHPg), has an active part in the inhibition of cell migration and invasion, by reducing cytoplasmic CD1 in breast cancer cells. We evidenced the existence of a CD1/Cdk4/Pxn axis that is the specific target of a ligand-activated PR-B signal, inhibiting Rac activity, thus we elucidated a novel mechanism regulating the mesenchymal-epithelial transition in breast cancer cells, further confirming the OHPg/PR-B protective effects in breast cancer.

## 2. Results

### 2.1. OHPg/PR-B Impair Aggressiveness of Breast Cancer Cells

**Figure 1:**
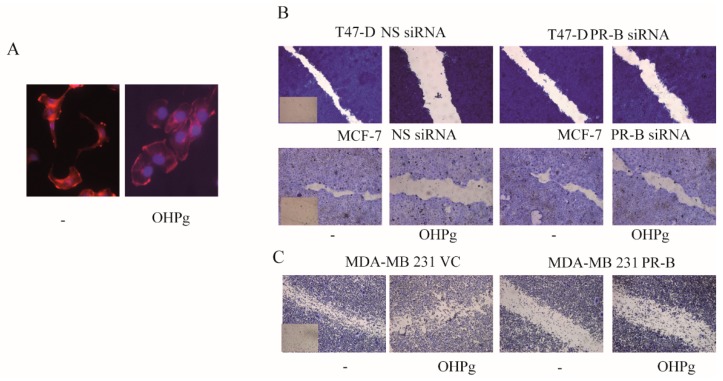

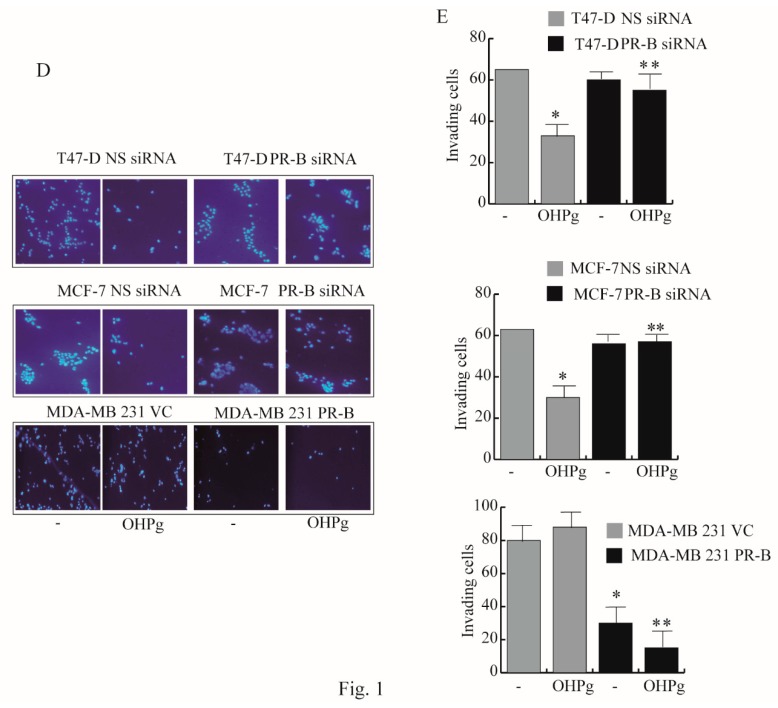
Hydroxyprogesterone (OHPg)-treated breast cancer cells show low motility, migration and invasion. (**A**) T47-D phalloidin staining of F-actin (stress fibers, red). 4′,6-diamidino-2-phenylindole (DAPI), nuclear staining. (**B**,**C**) Wound-healing assay (insets: time 0). T47-D and MCF-7 cells were transfected with non specific (NS) or targeted against Progesterone-Receptor (PR)-B siRNA. MDA-MB-231 were transfected with vector control (VC) or progesterone receptor B (PR-B) expression vector. (**D**) Transmigration assay, (**E**) Invasion assay. Columns are the mean of three independent experiments each in triplicate; bars, SD; * *p* ≤ 0.05 vs. vehicle treated cells. ** *p* ≤ 0.05 vs. OHPg-treated cells.

We first aimed to evaluate breast cancer cell morphology after 24 h (h) of 10 nM OHPg treatment. In these experimental conditions, Luminal A–type (T47-D) breast cancer cells, which revealed copy number gain of the PR gene [16], showed an increased adhesion with flatter, rounder, less elongated morphology (Figure 1A). Alongside, we observed an increased F-actin with a cortical distribution and a reduction in cytoplasmic microfilament bundling, compared with untreated cells. Next, to explore the OHPg effects in breast cancer cells’ motility, we performed wound-healing assays (Figure 1B). We found that OHPg-treated T47-D cells move much slower to close the gap compared with untreated cells, and this effect was greatly counteracted by specific PR-B siRNA. Similar results were obtained in MCF-7 cells, which revealed a heterozygous loss of the PR gene [16].To further analyze the OHPg/PR-B role in breast cancer cell motility, a PR negative, high motile MDA-MB-231 breast tumor cell line was transiently transfected with vector control (VC) or PR-B expression plasmids (Figure 1C). Consistently with the above-described results, we found that PR-B, exougenously expressed and/or activated by its native ligand, greatly impaired MDA-MB-231 motility, in wound-healing scratch assays. Next we investigated the capability of these cells to migrate across the uncoated membrane in transmigration assays, or to invade an artificial basement membrane Matrigel in invasion assays. T47-D- and MCF-7-untreated cells exhibited a migratory (Figure 1D and Supplementary Figure S1) and invasive (Figure 1E) behavior, which was significantly reduced by OHPg treatment; vector-expressing MDA-MB-231 cells showed a high migratory (Figure 1D and Supplementary Figure S1) and high invasive (Figure 1E) phenotype, and PR-B over-expression significantly reduced both migration and invasion. 

**Figure 2:**
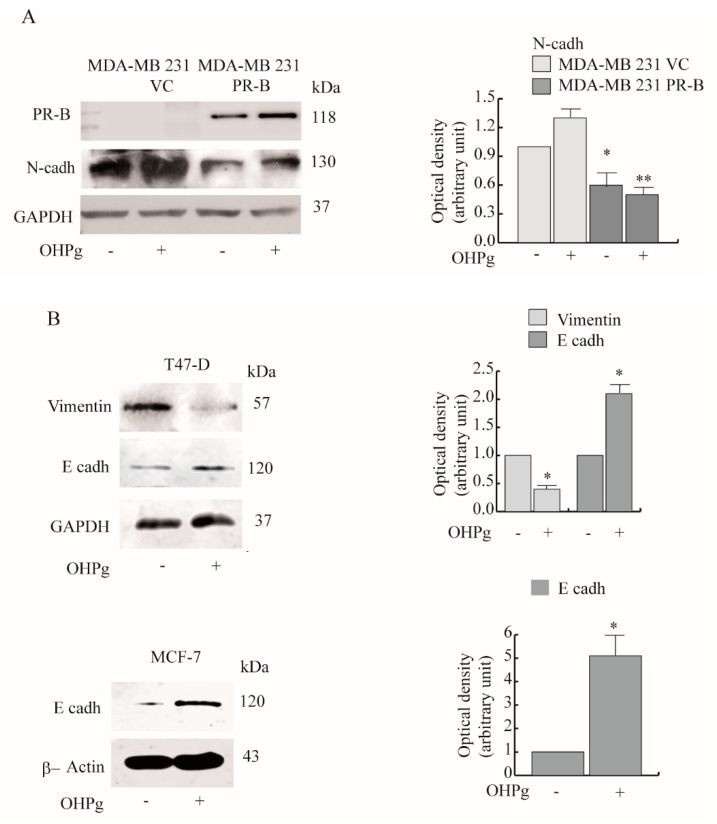
OHPg effects on N-cadherin (N-cadh), E-cadherin (E-cadh) and Vimentin expression in breast cancer cells. (**A**) Immunoblot analyses for PR-B and N-cadh expression. MDA-MB-231 cells transfected with vector control or PR-B expression vector were treated for 24 h, as indicated. Glyceraldehyde 3-phosphate dehydrogenase (GAPDH), control for loading. Columns refer to three independent experiments, as the mean of the band optical density expressed as fold over vehicle, which was assumed to be 1; bars, SD. * *p* ≤ 0.05 vs. vehicle-treated cells. ** *p* ≤ 0.05 vs. OHPg-treated cells. (**B**) Immunoblot analyses for Vimentin and E-cadh expression in T47-D and MCF-7 cells, as indicated. GAPDH and β-Actin, control for loading * *p* ≤ 0.05 vs. vehicle-treated cells.

N-cadherin (N-cadh) promotes cell motility [27], and it is highly expressed in MDA-MB-231. We observed that PR-B exogenous expression significantly reduced N-cadh levels, in the presence or absence of OHPg treatment (Figure 2A). Accordingly, OHPg treatment decreased the mesenchymal marker Vimentin in T47-D cells, as shown in Figure 2B upper panel (MCF-7 cells do not express Vimentin), alongside the epithelial marker E-cadh increased in both T47-D and MCF-7 cells (Figure 2B lower panel).

### 2.2. OHPg Decreases CD1 Expression Levels Through a Genomic Mechanism

**Figure 3:**
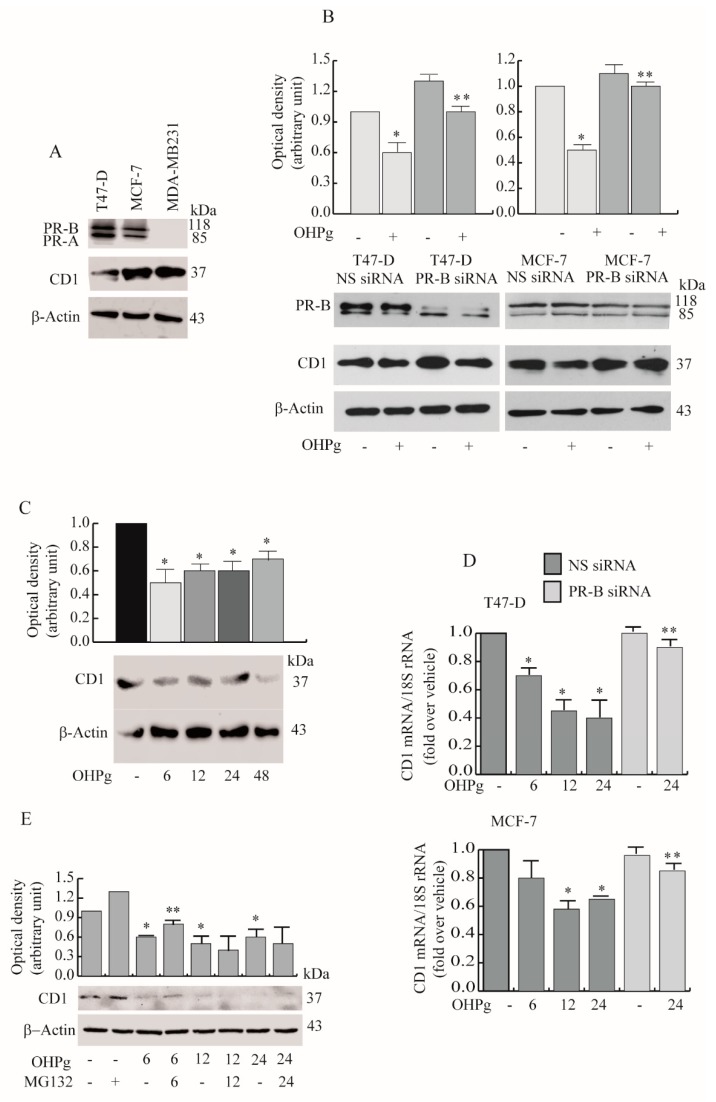
OHPg-treated breast cancer cells show a reduction of the cytoplasmic cyclin D1 (CD1) amount. (**A**) Immunoblot analyses for PR-B, progesterone receptor A (PR-A), CD1 expression in indicated cells and (**B**) in T47-D and MCF-7 cells transfected as indicated. Columns are the mean of three independent experiments in which CD1 band intensities were evaluated in terms of optical density arbitrary units, and expressed as fold over vehicle-treated NS siRNA cells, which was assumed to be 1; bars, SD. * *p* ≤ 0.05 vs. vehicle-treated NS siRNA cells. ** *p* ≤ 0.05 vs. OHPg-treated NS siRNA cells. (**C**) Immunoblot analyses for CD1 expression in MCF-7 cells treated at different times (h) as indicated by numbers. * *p* ≤ 0.05 vs. vehicle-treated cells. (**D**) Real-time polymerase chain reaction (PCR) assay of CD1 mRNA expression in T47-D (upper panel) and MCF-7 cells (lower panel), transfected and treated at different times as indicated. 18S rRNA was determined as the control. * *p* ≤ 0.05 vs. vehicle treated NS siRNA cells. ** *p* ≤ 0.05 vs. 24 h OHPg-treated NS siRNA cells. (**E**) Immunoblot analyses for CD1 expression.MCF-7 cells were pretreated with MG132 for 2 h and then co-treated with OHPg at different times (h) as indicated by numbers. * *p* ≤ 0.05 vs. vehicle-treated cells. ** *p* ≤ 0.05 vs. OHPg-treated cells.

To gain molecular insights into the biologic effects exerted by OHPg/PR-B on the migratory and invasive phenotype of breast cancer cells, we focused our interest onto Cyclin D1 (CD1), recently increasingly associated with metastasis in clinical studies and in vivo experiments [28]. Particularly, localization of CD1 in the membrane of fibroblasts and tumor cells has an active role in the induction of cell migration and invasion [13]. Cytoplasmic CD1 was detected in T47-D breast cancer cells, and in a greater extent in MCF-7 (Figure 3A). Notably, PR-negative high motile MDA-MB 231 breast cancer cells expressed much higher CD1 levels.

Next, we compared CD1 protein levels after 24 h of OHPg treatment in T47-D and MCF-7. Cytoplasmic CD1 expression decreased after OHPg stimulus, and the addition of a PR-B-targeting siRNA abrogated the OHPg-dependent down-regulation of CD1. PR-B siRNA also produced the increase of CD1 expression in untreated cells compared with NS siRNA cells, suggesting a ligand independent action of PR-B on CD1 expression (Figure 3B). OHPg induced similar effects in the nucleus (Supplementary Figure S2). Additionally, a time course study, performed in MCF-7 cells, evidenced a significant early (starting from 6 h of OHPg treatment) and sustained (until 48 h of OHPg treatment) decrease of cytoplasmic CD1 (Figure 3C). To establish if OHPg mediated the transcriptional or post-transcriptional regulation of CD1 expression, mRNA levels were evaluated by real time-PCR. As shown in Figure 3D, the mRNA levels of CD1 were significantly reduced after 6, 12 and 24 h of OHPg treatment in T47-D cells. Similar results were obtained in MCF-7 cells, although a faint CD1 mRNA decrease was observed at 6 h, suggesting OHPg effects on CD1 protein stability. To obtain evidence for the involvement of the cellular proteasome in OHPg action, the effects of the MG132 proteasome inhibitor on CD1 expression was examined in MCF-7 cells (Figure 3E). Pretreatment with 100 nM MG132 partially reversed the OHPg down-regulatory action of CD1 at 6 h, while no effect was evidenced later.

**Figure 4:**
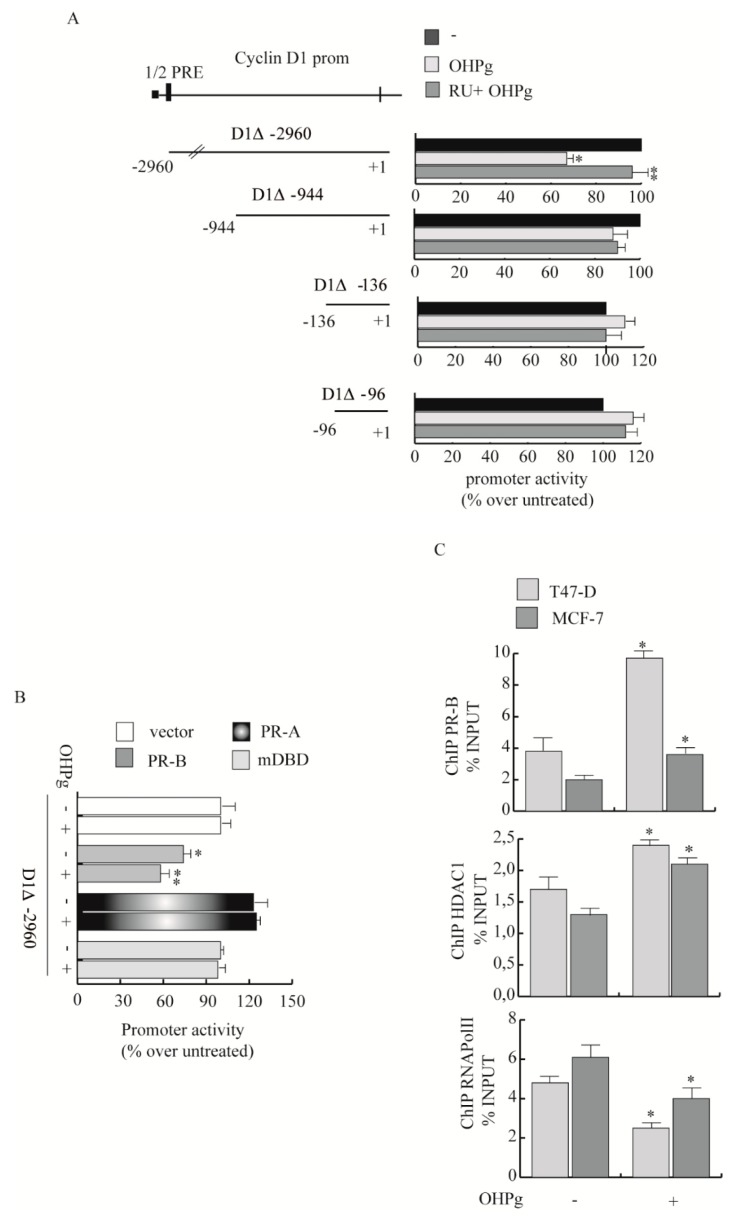
Effects of OHPg/PR-B on CD1 transcriptional activity. (**A**) Left panel: Diagram of the different CD1 gene promoter deletion constructs. Right panel: MCF-7 cells were transiently transfected, treated for 24 h with vehicle, 10 nM OHPg and 1µM RU 486, as indicated. Columns refer to three independent experiments expressed as fold change over vehicle, which was assumed to be 1; bars SD; * *p* ≤ 0.05 vs. vehicle. ** *p* ≤ 0.05 vs. OHPg. (**B**) MDA-MB-231 cells were co-transfected with vector control, D1Δ-2960 and PR-B or PR-A or DNA binding domain (mDBD) expression vectors, then treated as indicated; bars, SD; * *p* ≤ 0.05 vs. vector. ** *p* ≤ 0.05 vs. vehicle PR-B. (**C**) Chromatin Immunoprecipitation (ChIP)-qPCR. T47-D and MCF-7 cells treated with vehicle or OHPg for 6 h. Protein-DNA complexes were immune-precipitated with antibodies indicated. Columns are the mean of three independent experiments. Bars, SD; * *p* ≤ 0.05 vs. vehicle.

Then we explored the OHPg/PR-B action on CD1 gene transcription. To define PR responsive region(s) of the CD1 promoter, transient transfection studies were performed in MCF-7 by using the 5′ flanking region of the CD1 expression vector and three different deleted constructs (Figure 4A).

The construct, D1Δ-2960, which includes 2.960 kb of the CD1 promoter fragments, showed a marked decrease of transcriptional activity upon OHPg stimulation, with respect to untreated controls. Co-treatment with 1µM RU 486 (RU), a synthetic progesterone receptor antagonist [29], partially reversed the effect. Constructs D1Δ-944, D1Δ-136 and D1Δ-96 transcriptional activity was not significantly altered upon OHPg stimulation. These results suggest that in the region between −2960 bp to −944 bp are present regulatory elements involved in OHPg-mediated decrease of CD1 promoter activity. For instance, sequence analysis identified a canonical half-progesterone responsive element (half PRE) located from −2520 bp to −2510 bp.

To demonstrate PR-B contribution in the above-described effects, we co-transfected MDA-MB-231 cells with expression plasmids encoding either PR-B, PR-A or PR mutated in the DNA binding domain (mDBD). PR-B expression itself decreased the activity of D1Δ-2960, which was additionally reduced after OHPg stimulation. At the opposite, the PR-A isoform or mDBD had no effects (Figure 4B). Next we performed Chromatin Immunoprecipitation (ChIP) assays to demonstrate the specific recruitment of PR to the CD1 promoter region containing the half-PRE site that we identified within the CD1 promoter. Results obtained demonstrate that OHPg treatment caused an enhanced recruitment of PR, together with HDAC1 on the specific CD1 promoter region tested (Figure 4C), indicating that the chromatin could be in a less permissive environment for CD1 gene transcription, alongside which RNA Pol II was released. Altogether, these data strongly indicate that OHPg/PR-B represent fundamental down-regulators of CD-1 transcription.

### 2.3. OHPg Reduces the Interaction between CD1/Cdk4 and Paxillin in Vitro

**Figure 5:**
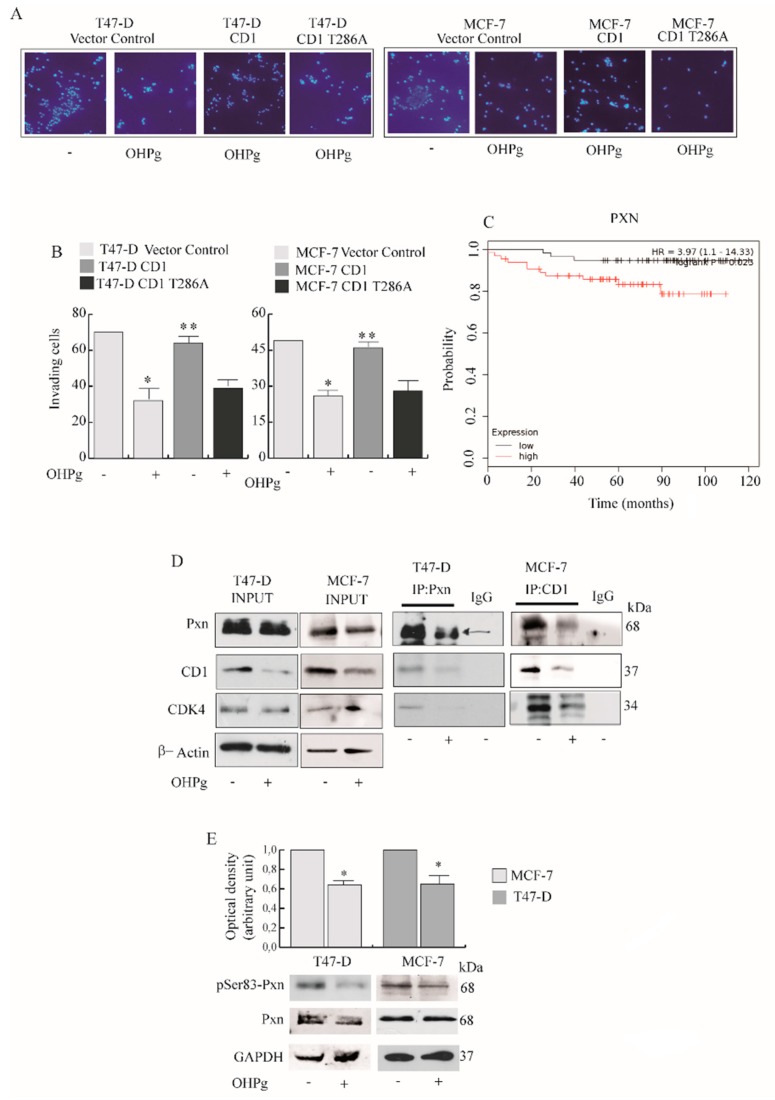
OHPg effects on CD1/Cdk4/Paxillin (Pxn) interaction and Pxn phosphorylation. (**A**) Transmigration assay, (**B**) Invasion assay. Cells were co-transfected with vector control, CD1 or phosphorylation site mutant of CD1 (CD1 T286A) expression plasmids. Columns are the mean of three independent experiments each in triplicate; bars, SD; * *p* ≤ 0.05 vs. vehicle treated vector control cells. ** *p* ≤ 0.05 vs. OHPg-treated vector control cells (**C**) Kaplan–Meier distant metastasis-free survival analysis in luminal A PR+ breast carcinoma patients (*n* = 122) with high and low Pxn expression analyzed as described in the Materials and Methods. Kaplan-Meier survival graph, and hazard ratio (HR) with 95% confidence intervals and logrank *p* value (**D**) Co-immunoprecipitation analysis (right panel). Cytoplasmic extracts were immunoprecipitated with anti-Pxn or anti-CD1 antibodies, as indicated, and immunoblotted with anti-Pxn, anti-CD-1 and anti-Cdk4. Input (left panel), samples without immunoprecipitation. βActin, loading control. IgG was used as the negative control. (**E**) Immunoblotting for pSer83 Pxn and Pxn expression in T47-D and MCF-7 cells, as indicated. GAPDH, loading control. Columns indicate the mean of relative ratio pSer83 vs. total Pxn. * *p* ≤ 0.05 vs. vehicle-treated cells.

Previous studies demonstrate that key components of focal adhesions (FAs) through the interaction with cytoplasmic CD1 may control cell migration and metastasis. Worthy of note, CD1 restoration (to original levels through transient exogenous expression) in OHPg-treated T47-D and MCF-7 cells, rescues the migratory (Figure 5A and Supplementary Figure S3) and invasive potential (Figure 5B), while CD1 T286A (mutated in phosphorylation site targeting CD1 for nuclear export) did not exert similar effects. Among FAs molecules, Paxillin (Pxn) is a multifunctional and multi-domain focal adhesion adapter protein, recruiting structural and signaling molecules involved in cell movement and migration [30]. The association among Pxn expression and distant metastasis free survival (DMFS) in Luminal A PR + breast cancer women (*n* = 122) was assessed by Kaplan–Meier analysis. Patients with high Pxn expression exhibited a lower rate of DMFS than those with low Pxn expression (*p* = 0.023), as illustrated in survival curves (Figure 5C). In our experimental conditions OHPg scantly reduced Pxn cytoplasmic levels (Figure 5D left panel). Next, we explored whether CD1 and its kinase partner Cdk4 could interact with Pxn. As shown in Figure 5D (right panel), we were able to detect co-immunoprecipitation of both endogenous cytoplasmic CD1 and Cdk4 with Pxn in basal conditions, and such complex formation was decreased in cells treated with OHPg.

Pxn is regulated by phosphorylation, and elevated protein phosphorylation was found in cancer tissues and metastatic cells, together with increased epithelial to mesenchymal transition [31]. Particularly, Pxn contains many putative phosphorylation sites, and it was demonstrated that Pxn serves as a substrate for the CD1/Cdk4 complex [13]. Thus, we analyzed the effects of OHPg on Pxn phosphorylation status. As shown in Figure 5E, OHPg treatment causes a reduction of *p*-Ser83 Pxn, the target of CD1/Cdk4 in both cell types. Interestingly, a reduction of *p*-Tyr118 Pxn, was evidenced in T47 D cells, but not in MCF-7 cells [32] (unpublished data).

### 2.4. OHPg/PR-B Impairs Breast Cancer Cell Migration through Pxn Phosphorylation Status

**Figure 6:**
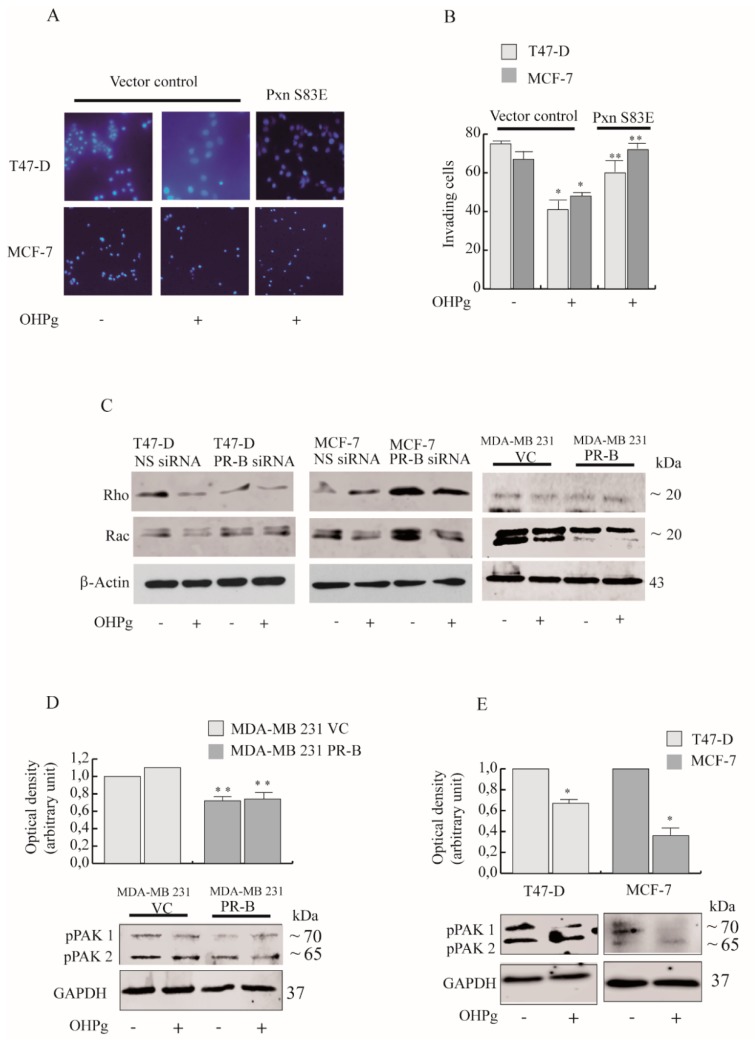
Phosphomimetic Pxn reverses the low invasive potential OHPg-treated cells. (**A**) Transmigration assay and (**B**) Invasion assay. Cells were co-transfected with a single phosphomimetic mutant of Pxn (Pxn S83E) or a vector control. Columns are the mean of three independent experiments each in triplicate; bars, SD; * *p* ≤ 0.05 vs. vehicle-treated cells. ** *p* ≤ 0.05 vs. OHPg-treated cells (**C**) Immunoblot analyses for Rho, Rac in T47-D, MCF-7 and in MDA-MB-231 cells transfected as indicated. β-Actin, loading control. Images show the results of one representative experiment out of three. (**D**) Immunoblot analyses for pPAK in MDA-MB-231 cells and in (**E**) T47-D, MCF-7 cells transfected as indicated. Columns are the mean of three independent experiments each in triplicate; bars, SD; * *p* ≤ 0.05 vs. vehicle-treated cells. ** *p* ≤ 0.05 vs. MDA-MB-231 VC vehicle-treated cells.

To demonstrate that OHPg/PR-B could negatively regulate breast cancer cell migration (Figure 6A and Figure S4) and invasion (Figure 6B) through the phosphorylation status of Ser83 in Pxn, we carried out functional assays with single phosphomimetic S83E (serine to glutamic acid) Pxn mutants. Under our assay conditions, the single phosphomimetic S83E rescues the migratory and invasive potential of T47-D and MCF-7 cells.

Pxn, interacts with numerous molecules thus controlling the Rho family of GTPases, crucial regulators of adhesion dynamics [33]. Rac1 GTPase is the major inductor of membrane ruffling and is required for cell migration [34]. Previous studies demonstrate that CD1/Cdk4 via Pxn phosphorylation at Ser83, interferes with Rac1 activity [13]. Since OHPg inhibits Pxn phosphorylation, we postulated that OHPg/PR-B could alter Rac activity and cell invasion. In our experimental conditions Rac 1,2,3 expression levels appear substantially decreased by OHPg/PR-B in T47-D cells, while in MCF-7 the effects of OHPg appear to be only partially dependent by PR-B. In MDA-MB231 cells, PR-B transient transfection causes a ligand independent down-regulation of both detected bands. Instead, RhoA/B/C levels were not uniquely modulated by OHPg/PR-B in the three cell lines tested. (Figure 6C and Figure S4).

In the GTP-bound activated form, Rac1–3 proteins are able to interact with p21-activated kinase (PAK1) and to stimulate its in vitro autophosphorylation at serine 144 [35], leading to the activation and stabilization of filamentous actin structures [36].

In MDA-MB 231 exogenously expressing PR-B, pSer144–PAK1 levels were greatly reduced compared with MDA-MB-231 VC cells (Figure 6D). Furthermore, in OHPg-treated T47-D and MCF-7 cells, the levels of pSer144-PAK1 are decreased (Figure 6E) and the single phosphomimetic S83E counteracted this effect (unpublished data) [32], indicating that OHPg through Ser83 in Pxn regulates the activity of Rac1.

## 3. Discussion

There are controversial evidences regarding the functional role of ligand-activated PRs in breast cancer cell aggressiveness. Recently, McFall et al. [37] demonstrated that higher doses of progesterone or synthetic progestins induce invasiveness, while lower doses of progesterone within the physiological range does not exert stimulatory effects. Further elegant studies [38] have elucidated a critical role for the short PR isoform A in enabling the progestagen R5020 to oppose specific actions of estrogen, thus promoting the invasiveness and metastasis of breast cancer cells.

However, the Progesterone receptors’ action is highly context- and cell type-dependent, but also heavily influenced by post-translational modifications. Several evidences suggest a progesterone inhibitory action in cell migration which relies upon sustaining mechanisms for cell-cell interaction and cell adhesion, maintaining the epithelial integrity [39,40]. Besides, clinical data suggest that PR status influences metastatic spread with notable differences in survival after relapse of breast cancer subtypes. For instance, recent data report that PR negative, luminal A subtype, has the higher risk of metastasis, especially late recurrence, than the PR positive, luminal A subtype, indicating that PR expression and tumor size were independent prognostic factors in the luminal A-like subtype [41]. Further studies establish that PR absence is a negative prognostic factor in breast cancer patients, with ER-positive locoregional recurrence [42]. Moreover, it is reported that ER-positive/PR-negative tumors display more invasive features than ER-positive/PR-positive tumors, despite higher levels of HER-1 and HER-2 [43].

The lack of both PRs isoforms’ expression in ER-positive tumors is indicative of aberrant growth factor signaling, contributing to breast cancer recurrence and metastasis. Conversely, high levels of PRs, in breast cancer cell models, are related to progestins-induced expression of desmoplakins [44], which interact with transmembrane linker proteins to hold the adjacent membranes together.

Nevertheless, the effects of an imbalance in the native ratio of A to B forms of PR, as well as the distinct role of the two PR isoforms in breast cancer progression, is still to be defined. In this concern in vivo studies report that the mammary glands of transgenic mice carrying altered PR-A/PR-B ratio exhibited decreased cell-cell adhesion [45]. Herein, we show that OHPg/PR-B evoke the reverse of a motile and invasive phenotype of luminal A breast cancer cells, inducing the so called mesenchymal–epithelial transition. OHPg reduces Vimentin, the major intermediate filament in mesenchymal cells, while it induces E-cadh, a trans-membrane protein epithelial origin involved in the strength of cellular adhesion within a tissue. Moreover, in our experimental conditions, different than those used by McFall et al., PR-B exogenous expression is sufficient to decrease the migratory and invasive potential of high-invasive triple-negative MDA-MB-231 cells, in agreement with studies reporting that steroid receptors exhibit ligand-independent activation under appropriate conditions [46]. MDA-MB-231 cells expressing lower amounts of PR-B showed OHPg-dependent effects on cell invasion [32] (unpublished data).

Our study demonstrates that the molecular mechanisms by which the PR-B isoform, activated by its own natural ligand, impairs migration and invasion, is crucially dependent by the regulation of the cytoplasmic CD1 amount, although at present we do not investigate the impact on metastasis. Particularly, the restoration of CD1 expression to the original levels rescues the migratory and invasive potential, while CD1 T286A (mutated in the phosphorylation site targeting CD1 for nuclear export) did not. Cyclins were absolutely considered as nuclear proteins, regulating cell cycle transitions [47]. Nevertheless, emerging data establish that these cell cycle molecules are located in the cytoplasm where they regulate different cell functions. Recent studies demonstrated the functional and physical interaction of CD1 with cytoplasmic and membrane-associated proteins, indicating that this cyclin could play an active role regulating adherence and migration [28].

Herein we demonstrate that OHPg/PR-B cause the reduction of CD1 amount by a genomic mechanism. By sequence analysis we identified a half PRE-site at the CD1 promoter and ChIP assay which further confirmed that OHPg treatment induced the binding of PR-B to the identified responsive sequence. Alongside, the recruitment of HDAC1 indicates a less permissive chromatin conformation for gene transcription, confirmed by the release of RNA Pol II. These findings corroborate our previous data, demonstrating that PTEN, which inhibits CD1 levels and nuclear activity [48], is a target of OHPg/PR-B protective effects in breast cancer cells [49]. 

These data are in agreement with in vivo evidence [50] reporting a reduction in the percentage of PR-positive cells following PTEN loss in the luminal compartment of the adult mammary gland.

Cyclin D1 and its kinase partner Cdk4 play the best studied role as regulator of transcription in the nucleus [8,51]. Instead, only several authors proposed their cytoplasmic biological functions. The increase of CD1, together with Cdk4 outside the nucleus, was initially described as a mechanism for the cell cycle arrest [52]. Our data show that OHPg produces a CD1 decrease in the cytoplasm, but also in the nucleus, consistent with our previous data reporting that OHPg acting through PR-B decreases E2-induced cell proliferation in breast cancer cells [53]. Interestingly, very recent acquisitions show that the localization of CD1 outside the nucleus, in the membrane may affect cell migration and invasion of fibroblasts and tumor cells [11,12]. These findings propose a new mechanism by which CD1 through Cdk4 controls the phosphorylation of a subpopulation of cytoplasmic Pxn molecules, which provide docking sites for the assembly of multiprotein complexes acting on cell-matrix adhesion and cell migration.

Very few published data suggest a potential action of progesterone on Pxn expression levels, although a recent study reports that mifepristone, a progestational and glucocorticoid hormone antagonist, inhibited the expression of Pxn in MDA-MB-231 cells [54]. Here, using Kaplan-Meier analysis we found that low Pxn expression was associated with increased distant metastasis-free survival in luminal A PR+ breast carcinomas, suggesting its potential role as a prognostic marker. We show a scant reduction of total Pxn by OHPg stimulus. Interestingly, the significant decrease of CD1 located in the cytoplasm, due to OHPg/PR-B action, affects the functional amount of CD1 interacting with the Cdk4 and available for Pxn interaction and phosphorylation [13]. The regulation of Pxn through phosphorylation is reported [31]. In particular, CD1/Cdk4-mediated phosphorylation of Pxn at Ser 83 is essential for the modulation of cell spreading and invasion in vivo. Our results indicate that OHPg causes a reduction of Pxn phosphorylation at Ser 83. Remarkably, the reduced phosphorylation at Ser 83 is essential for OHPg/PR-B effects on migratory and invasive phenotypes of breast cancer cells. Indeed, the single phosphomimetic S83E rescues the migratory and invasive potential of T47-D and MCF-7 cells, despite OHPg action.

Cyclin D1 binds to the C-terminal region (LIM domains) of Pxn [13], and LIM domains are required for the efficient targeting of Pxn to FAs [31]. Both interactions could be mutually exclusive, therefore we can reasonably retain that upon OHPg stimulus, the Pxn amount dissociated from CD1 interaction could localize at FAs to control cell adhesion. Previous data indicates that Pxn located at FAs may lead to more efficient cell spreading, while Pxn phosphorylation by CD1 at the cell membrane may lead to an opposite effect. In this concern, Y31/118-phosphorylated Pxn is present at different locations, promoting different effects on cell adhesion [55].

Membrane ruffling and the protrusive activity of cells is strictly regulated by Rac1. For instance Rac1−/− fibroblasts are compromised in migration as CD1−/− cells [13]. Enhanced migration and invasiveness results in the hyperactivation of the Rac pathway in cancer. Our results demonstrate that low Pxn phosphorylation levels at Ser 83, consequent to the reduced CD1/Cdk4 functional interaction, led to inhibition of Rac1-activity, as evidenced by the decrease of pSer144-Pak1 levels, in all breast cancer cell lines tested. Indeed overexpression of the single phosphomimetic S83E, counteracted OHPg effects on pSer144-Pak1 levels.

## 4. Materials and Methods

### 4.1. Reagents

17-Hydroxyprogesterone (OHPg), aprotinin, leupeptin, phenylmethylsulfonyl fluoride (PMFS), sodium orthovanadate, NaCl, MgCl2, EGTA, glycerol, Triton X-100, Fetal Calf Serum (FCS), Fetal bovine serum (FBS), HEPES were from Sigma-Aldrich (Milan, Italy). Antibodies against human Progesterone-Receptor (PR), HDAC1, RNA Pol II, Cyclin D1, Cdk4, paxillin (Pxn), *p*-Tyr118 Pxn, *p*-Ser83 Pxn, E-cadherin (E-cadh), N-cadherin (N-cadh), Vimentin, glyceraldehyde 3-phosphate dehydrogenase (GAPDH), β-actin and Protein A/G PLUS-Agarose were from Santa Cruz Biotechnology (Santa Cruz, CA, USA) Rac1–3, RhoA-C (Thermo Fisher, Waltham, MA, USA), *p*-Ser144 PAK1/pSer141 PAK2(Cell Signaling, Danvers, MA, USA). RU 486 and MG132 were from Calbiochem (Milan, Italy).

### 4.2. Plasmids

D1Δ-2960, D1Δ-944, D1Δ-136 and D1Δ-96, carrying fragments from the human cyclin D1 promoter and inserted into the luciferase vector pXP2 (a gift from Dr A. Weitz, University of Naples, Napoli, Italy). The full-length progesterone receptor B (PR-B) [12] consisting of the full-length PR-B cDNA fused with the SV40 early promoter and expressed in the pSG5 vector (a kind gift from Dr. D. Picard, University of Geneva, Geneva, Switzerland); the full length progesterone receptor A (PR-A) (a gift from Prof. Paul Kastener (Laboratory of Molecular Genetics, CNRS, Strasbourg, France). PR DNA-binding mutant C587A (DNA binding domain (mDBD) PR) was kindly provided by Dr. C. Lange (University of Minnesota Cancer Center, Minneapolis, MN, USA) [11]. pcDNA cyclin D1 HA and pcDNA cyclin D1 HA T286A were from Addgene. The single phosphomimetic mutant of Pxn (PxnS83E) [13] was kindly provided by Dr. E. Gary (Cell Cycle Lab, Institut de Recerca Biomèdica de Lleida (IRBLleida), and Departament de Ciències Mèdiques Bàsiques; Facultat de Medicina; Universitat de Lleida, 25,198 Lleida, Catalonia, Spain). The Renilla luciferase expression vector pRL-TK (Promega, Milan, Italy) was used as a transfection standard.

### 4.3. Cell Culture

T47-D, MCF-7 and MDA-MB-231 human breast cancer cells were obtained and authenticated from the American Type Culture Collection (Manassas, VA, USA), stored according to the supplier’s instructions, and used within four months after frozen aliquot resuscitations. T47-D cells were routinely maintained in RPMI 1640, supplemented with 5% FCS, 1μg/mL insulin (Sigma, Milan, Italy), 1 mg/mL penicillin/streptomycin (Sigma, Milan, Italy). MCF-7 were maintained in DMEM/F-12 medium containing 5% FCS, 1% L-glutamine, 1% Eagle’s nonessential amino acids, and 1 mg/mL penicillin/streptomycin in a 5% CO_2_-humidified atmosphere [56] MDA-MB-231 were maintained in DMEM/F-12 medium containing 5% FBS. Mycoplasma negativity was tested monthly (MycoAlert, Lonza, Walkersville, MD, USA). After serum starvation for 24 h, cells were treated in medium containing 5% charcoal-treated FCS, to reduce the endogenous steroid concentration, using 10 nM OHPg, for different times, as indicated.

### 4.4. Total RNA Extraction, Reverse Transcription PCR and Real-Time RT-PCR Assay

Total RNA was extracted from T47-D and MCF-7 cells using TRIzol reagent and cDNA was synthesized by the reverse transcription-polymerase chain reaction (PCR) method using a RETROscript kit [57] Five microliters of diluted (1:4) cDNA was analyzed using SYBR Green Universal PCR Master Mix, following the manufacturer’s recommendations. Primers used for the amplification were 5′-CGTGGCCTCTAAGATGAAGGA-3′ (forward) and 5′-CGGTGTAGATGCACAGCTTCTC-3′ (reverse). Real-time PCR was performed in the iCycler iQ Detection System (Bio-Rad, Milan, Italy), using 0.1 μM each primer in a total volume of 30 μL of reaction mixture following the manufacturer’s recommendations. Each sample was normalized on the basis of its 18S ribosomal RNA content. The results were calculated and expressed as previously reported [21].

### 4.5. Immunoprecipitation and Western Blot

Cells were exposed to treatments for different times and processed to obtain cytoplasmic or nuclear fractions, as previously described [58]. Immunoprecipitation was performed as previously described [49]. Briefly, 500 µg of cytoplasmic protein lysates were incubated overnight with the specific antibody and 500 µL of HNTG buffer 50 mmol/L HEPES (pH 7.4, slightly alkali), 50 mmol/L NaCl, 0.1% Triton X-100, 10% glycerol, 1 mmol/L phenylmethylsulfonyl fluoride, 10 Ag/mL leupeptin, 10 Ag/mL aprotinin]. Immunocomplexes were recovered by incubation with protein A/G-agarose. 

The beads containing bound proteins were washed by centrifugation in immunoprecipitation buffer, then denatured by boiling in Laemmli sample buffer and analyzed by Western blot. Autoradiographs show the results of one representative experiment out of three. The band intensity was evaluated by densitometry using the Scion Image 4.0.3.2 software.

### 4.6. Transient Transfection and Luciferase Assays

Transient transfection studies were performed as described [59]. Cells were transfected using the FuGENE 6 or Lipofectamine 2000 reagent (Invitrogen, Paisley, UK) as recommended by the manufacturer, with a mixture containing specific constructs. Cells were incubated for 24 h after treatments. Renilla luciferase plasmid (25 ng/well) was used as standard luciferase assays. Firefly and Renilla luciferase activities were measured using a Dual Luciferase Kit (Promega, Milan, Italy)

### 4.7. Lipid-Mediated Transfection of siRNA Duplexes

Cells were transfected with 4 functionally-verified siRNA directed against human PR-B or with a control siRNA (Qiagen, Milan, Italy) that does not match with any human mRNA used as a control for non-sequence specific effects. Cells were transfected [60] using Lipofectamine 2000 reagent (Invitrogen, Paisley, UK) and then treated as indicated.

### 4.8. Chromatin Immunoprecipitation (ChIP) Assays and Realtime ChIP

Cells were treated for 6 h, then DNA/protein complexes were extracted as described [61]. The precleared chromatin was immunoprecipitated with specific antibodies against PR, HDAC1 and RNA Pol II, as indicated. Normal rabbit IgG (Cell Signaling, #2729) was used instead of primary Ab as negative control. Immunoprecipitated DNA was analyzed in triplicates by real-time PCR by using 5 µL of the diluted (1:3) template. The following primers, corresponding to the cyclin D1 (CD1) promoter region containing the half PRE site, were used: Forward 5′-CCAAGAAATAAGAACAGAGCAC-3′ and reverse 5′-CTTTTCGGTTGCAGTTTTAC-3′. Input DNA quantification was performed by using 5 μL of the diluted (1/50) template DNA. Final results were calculated as previously described [61].

### 4.9. Wound-Healing Assays

The method was performed as previously described [62]. Confluent cell monolayers were scraped and subjected to the various experimental conditions. Wound closure was monitored at different times (T47-D and MCF-7 18 h, MDA-MB231 12 h), then cells were fixed and stained with Coomassie Brilliant Blue (triphenylmethane dye). Pictures represent one of three independent experiments (10× magnification, phase-contrast microscopy).

### 4.10. Transmigration Assays

This method was performed as previously described [63]. Cells from the various experimental conditions were placed in the top compartments of Boyden chambers (8-μm membranes, Corning). The bottom well contained regular growth media. After 12 h (T47-D and MCF-7) and 8 h (MDA-MB 231), migrated cells were fixed and stained with 4′,6-diamidino-2-phenylindole (DAPI). Migration was quantified in five separate fields/membrane (10× magnification) and expressed as the mean of migrated cells. Data represent three independent experiments, assayed in triplicate.

### 4.11. Invasion Assays

Matrigel-based invasion assay was performed in Boyden chambers (8-μm membranes) coated with Matrigel (BD Biosciences, 2 mg/mL). Cells were exposed to various experimental conditions for 48 h and then placed in top compartments. The bottom well contained regular growth media containing 10% FBS. After 12 h (T47-D and MCF-7) and 8 h (MDA-MB 231), invaded cells were quantified as reported for transmigration assays.

### 4.12. Phalloidin Staining

Polymerized actin stress fibers were stained with Alexa Fluor 568–conjugated phalloidin, following the manufacturer’s instructions (Life Technologies, Milan, Italy). Cell nuclei were counterstained with DAPI. An Olympus BX51 microscope (100× magnification) was used for imaging.

### 4.13. Statistical Analysis

The data were analyzed by Student’s t test using the GraphPad Prism 4 software program and the results were presented as mean ± SD. A value of *p* ≤ 0.05 was considered to be significant. Kaplan-Meier analysis was performed as described [64]. Samples were from 122 patients (ER status all, PR status positive, Her2 status all, intrinsic subtype luminal A). Kaplan-Meier survival graph, and hazard ratio with 95% confidence intervals and logrank *p* value were calculated using Kaplan Meier plotter.

## 5. Conclusions

**Figure 7:**
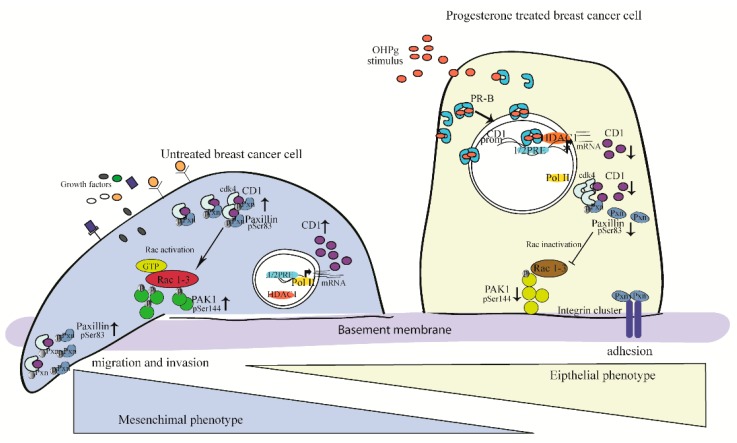
Proposed model for OHPg/PR-B-induced mesenchymal-epithelial transition in breast cancer cells. See text for details.

The most convincing interpretation of our results is that OHPg/PR-B, by a genomic mechanism, reduces the cytoplasmic levels of the functional CD1 amount. The latter recruits less Cdk4 and Pxn, which appears consequently less phosphorylated, thus able to sustain cell adhesion (Figure 7). At the present, the cyclin D/Cdk4,6 complexes are considered relevant targets for cancer therapy. Our data are consistent with an onco-suppressor model in which OHPg/PR-B act as novel inhibitors of CD1/Cdk4, thus promoting the mesenchymal-epithelial transition and the reduction of breast cancer cell aggressiveness. Future studies are focused in evaluating the possibility of combining this agent with existing therapies for advanced breast cancer.

## Supplementary Materials

The following are available online at https://www.mdpi.com/2072-6694/11/8/1201/s1, Figure S1: Transmigration assay, Figure S2: Immunoblot analysis, Figure S3: Transmigration assay, Figure S4: Transmigration assay and Immunoblot experiments.
