# Supplementary material for: Progesterone Receptor B signaling Reduces Breast Cancer Cell Aggressiveness: Role of Cyclin-D1/Cdk4 Mediating Paxillin Phosphorylation
Source: Cancers (Basel). 2019 Aug 17;11(8):1201. doi: 10.3390/cancers11081201 (PMC6721542; doi:10.3390/cancers11081201)
Supplement: Supplementary file 1 [file cancers-11-01201-s001.pdf]

Article

Progesterone Receptor B signaling Reduces Breast Cancer Cell Aggressiveness: Role of Cyclin-D1/Cdk4 Mediating Paxillin Phosphorylation

Francesca Ida Montalto, Francesca Giordano, Chiara Chiodo, Stefania Marsico, Loredana Mauro, Diego Sisci, Saveria Aquila, Marilena Lanzino, Maria Luisa Panno, Sebastiano Andò and Francesca De Amicis

Supplementary Materials

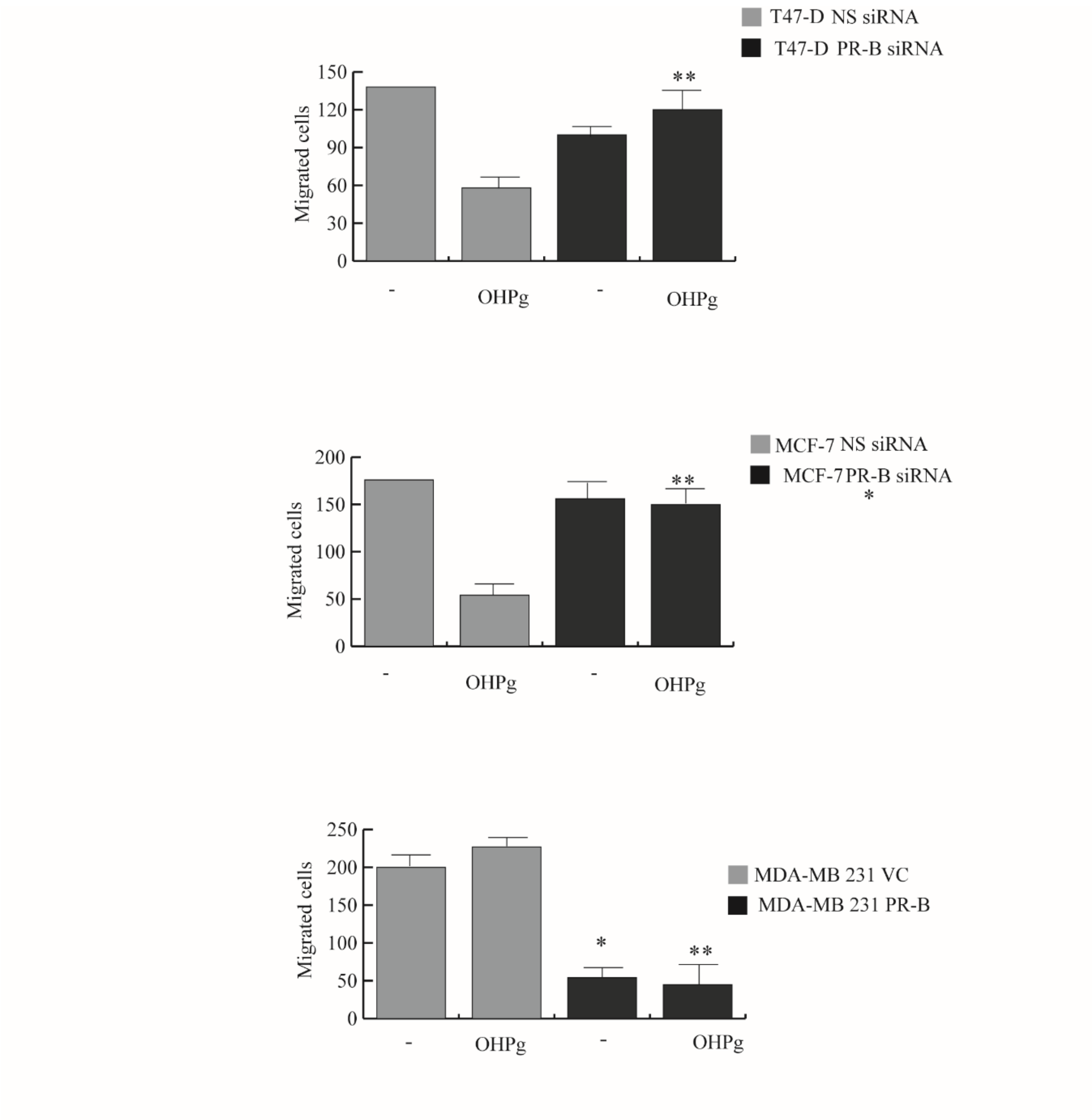

Figure S1. Transmigration assay. Columns are the mean of three independent experiments each in triplicate represented in Figure 1D; bars, SD; * $p \leq 0.05$ vs vehicle treated cells. ** $p \leq 0.05$ vs. OHPg treated cells.

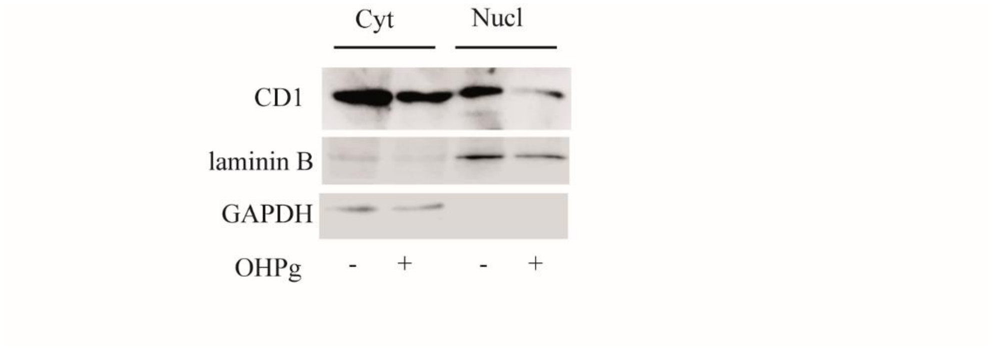

Figure S2. Immunoblot analyses for CD1 expression in MCF-7 cells treated as indicated. Laminin B and GAPDH are used as loading control.

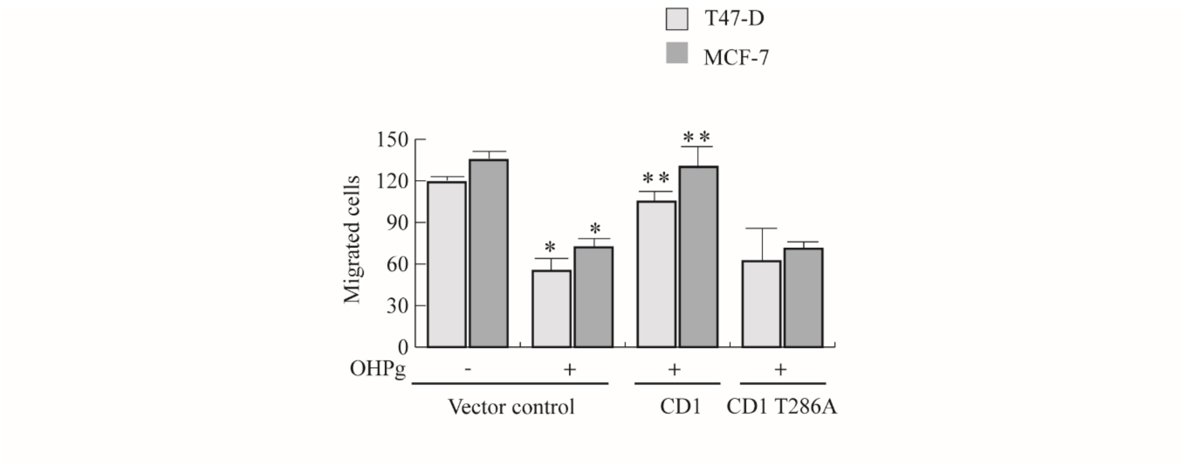

Figure S3. Transmigration assay. Columns are the mean of three independent experiments each in triplicate represented in Figure 5A; bars, SD; * $p \leq 0.05$ vs. Vector control vehicle treated cells. ** $p \leq 0.0$ vs. Vector control OHPg treated cells.

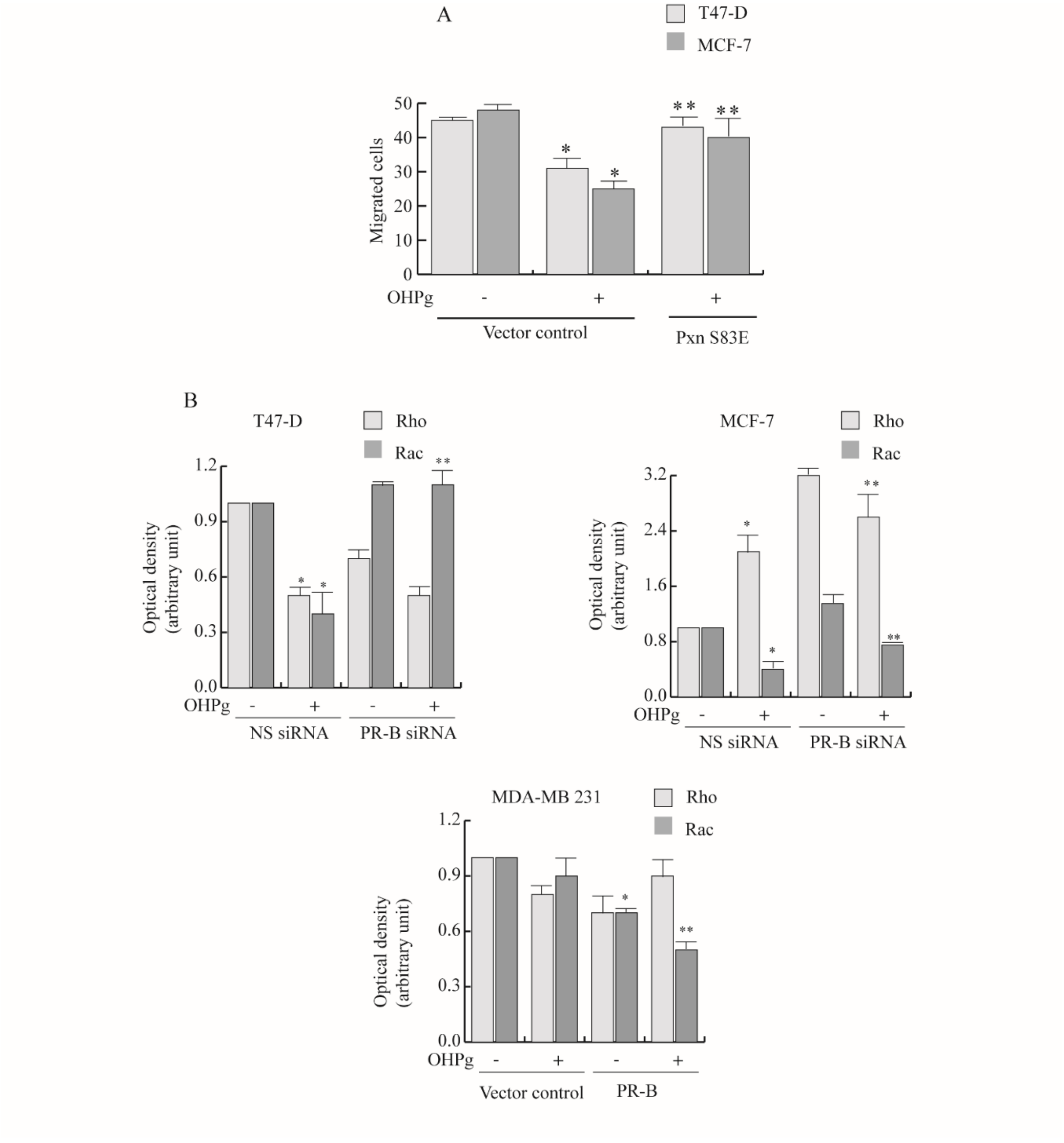

Figure S4. Transmigration assay and Immunoblot experiments. (**A**) Transmigration assay. Columns are the mean of three independent experiments each in triplicate represented in Figure 6A; bars, SD; * $p \leq 0.05$ vs. vehicle treated cells. ** $p \leq 0.05$ vs. OHPg treated cells. (**B**) Immunoblot experiments represented in Figure 6C. Columns, are mean of three independent iin which Rho, Rac band intensities were evaluated in terms of optical density arbitrary units and expressed as fold over vehicle treated NS siRNA or Vector control cells, which was assumed to be 1; bars, SD. * $p \leq 0.05$ vs. vehicle treated NS siRNA or Vector control cells. ** $p \leq 0.05$ vs. OHPg treated NS siRNA or Vector control cells.

© 2019 by the authors. Licensee MDPI, Basel, Switzerland. This article is an open access article distributed under the terms and conditions of the Creative Commons Attribution (CC BY) license (http://creativecommons.org/licenses/by/4.0/).
